# Scale-Compatible Aboveground Biomass Estimation Using Canopy Cover: Evidence from the Gobi Desert of Northwest China

**DOI:** 10.3390/plants15213444

**Published:** 2026-11-05

**Authors:** Hanjian Hu, Lingchao He, Rujie Ye, Kang Xu

**Affiliations:** 1 College of Urban Construction, Zhejiang Shuren University, Shaoxing 312028, China; huken@zjsru.edu.cn (H.H.); 202302316119@stu.zjsru.edu.cn (R.Y.); 2 College of Life Sciences, Zhejiang University, Hangzhou 310058, China; 3 Jiangsu Key Laboratory for Recognition and Remediation of Emerging Pollutants in Taihu Basin, School of Environmental Science and Engineering, Wuxi University, Wuxi 214105, China

**Keywords:** aboveground biomass estimation, multi-scale, canopy cover, Gobi Desert

## Abstract

Multiple morphological factors of desert plants were employed to construct aboveground biomass (AGB) estimation models in the Gobi Desert, located in the northwestern part of Gansu Province, China, where gravel surfaces account for more than half the area. At the individual scale, estimation models incorporating variables related to canopy cover accurately estimated AGB (R^2^ = 0.623~0.848). At the quadrat scale, the cover-allometric model effectively estimated multi-species AGB (AGB = 0.302 × C_t_^0.900^, R^2^ = 0.725). At the regional scale, the model incorporating relative canopy cover was effective for estimating aboveground biomass density (AGB_D_) and could be combined with regional area estimates to determine the AGB of desert plants at any spatial scale (AGB_D_ = 20.114 × C_r_^1.043^, R^2^ = 0.8313). AGB estimation models based on canopy cover not only overcome scale limitations but also exhibit a tendency toward improved model fit with increasing scales. However, when applied to remote-sensing data, the model exhibits relatively low fitting accuracy (AGB_D_ = 4.398 × C_r_^0.538^, R^2^ = 0.3656). It demonstrates good stability when applied to Gobi Desert habitats similar to the study area, but its applicability in other arid regions still requires further validation.

## 1. Introduction

Biomass typically denotes the aggregate quantity of organic matter per unit area of biological components within an ecosystem. It serves as a fundamental quantitative characteristic of plants and directly reflects the production potential of ecosystems [1,2]. Biomass is also associated with carbon storage, rendering it a vital metric for assessing the carbon sequestration capacity of plant communities [3]. Owing to the significance of issues such as global change, carbon cycling, carbon sink losses, and carbon trade, biomass estimation persists as a significant research topic [4]. The dynamic alteration of plant aboveground biomass (AGB) directly reflects the interrelationship between plants and environmental variables. It is the most attainable and direct indicator for understanding ecosystem functioning [5,6].

Numerous AGB estimation models exist for ecosystems like forests [7], while the Gobi Desert receives relatively little attention, owing to low biomass density in arid regions. Nevertheless, the Gobi Desert, spanning an area of 660.8 thousand square kilometers, accounts for 6.88% of the total land area of China [8]. The Gobi Desert represents crucial terrestrial ecosystems; the plant communities in the Gobi Desert exhibit high resistance to drought, cold, water erosion, wind erosion, and soil infertility, among other factors. This attribute endows them with far-reaching significance for the sustainable development and ecological construction of Gobi Desert ecosystems [9,10]. The estimation of the AGB of vegetation in the Gobi Desert facilitates the evaluation of terrestrial carbon sequestration, which holds significant importance for desertification monitoring and mitigating global climate change [6,9,11].

The quantification of plant biomass predominantly includes direct harvesting, remote-sensing techniques, photographic methods, and modeling methods [4,9]. Although the direct harvesting method offers precise estimations of AGB, it may cause potential damage to fragile ecosystems like deserts [12,13]. Moreover, direct harvesting is both time-consuming and labor-intensive when attempting to obtain a sufficient number of samples to meet statistical requirements [14]. Non-destructive sampling methods represented by the partial harvesting method are widely adopted for estimating the biomass of desert plants, in which the total biomass of each plant is estimated by harvesting partial tissue samples from target individuals [15,16]. Desert vegetation typically exhibits low-stature, indistinct spectral characteristics, and fine-scale spatial heterogeneity [17,18,19], and satellite images are not sufficient to provide the spatial resolution to identify the characteristics of Gobi Desert communities [20,21,22]. Therefore, remote-sensing techniques are also limited in such environments [20,23,24]. The photographic method has met the demand for high spatiotemporal resolution during an expansion of satellite remote-sensing data [9,25]. The introduction of unmanned aerial vehicles (UAVs) enables the collection of high-resolution data regarding the spectral and structural properties of low-stature vegetation at fine scales [26,27]. Images obtained by UAVs facilitate the efficient identification and extraction of vegetation characteristics from the soil background, and UAV images are rarely distorted by cloud cover [28,29]. However, the sparse vegetation and gravel-covered surface in the Gobi region introduce systematic biases in UAV-based vegetation identification. Certain plant species, characterized by slender branches and susceptibility to burial by sand, are prone to detection omission; this phenomenon is further exacerbated during non-growing seasons due to increased leaf litter [30]. Furthermore, the spectral characteristics of gravel-covered surfaces overlap with those of some desert plants, further contributing to systematic biases, while mixed pixels comprising canopy-edge vegetation and bare soil further reduce identification accuracy [9,31]. In addition, flight height is a crucial parameter, as it affects spatial resolution of images [32,33,34], and UAV-based techniques do not always accurately capture biomass-related indicators at any flight height [35]. The combination of remote sensing and technology-based photographic methods can provide ultra-high-resolution images for large-scale vegetation AGB estimation [36,37], but it is more demanding with respect to operation and experience, and is generally impractical for ecological research in terms of convenience. The accuracy of the aforementioned biomass quantification methods should be verified based on models of field survey data. As a simple yet effective method for large-scale biomass estimation, the modeling method substantially reduces environmental damage and exhibits high feasibility for extensive application [12,38,39]. However, in comparison with other ecosystems like forests, the utilization of modeling methods in desert ecosystems has received limited attention, and further optimization is required to adapt to desert environments [9]. Species-specific allometric models have been developed for dozens of dominant desert species. However, substantial interspecific divergence in life forms, lignification degrees, and branching architectures leads to limited model generalizability. Consequently, single-species models cannot be directly applied to multi-species communities, and no integrated modeling framework has been established that concurrently accommodates species-specific traits and community-level applicability [40]. Furthermore, the scaling extrapolation mechanisms underlying these models remain poorly elucidated. During the extrapolation of high-precision individual-scale allometric models to larger scales, results are confounded by the synergistic impacts of spectral interference from stony Gobi surfaces, mixed-pixel effects of sparse vegetation, and species-level spatial heterogeneity. This leads to pronounced scale mismatches between field-measured parameters and remote-sensing inversion results. A robust cross-scale estimation framework is still unavailable, and research specifically addressing such extreme habitats remains comparatively scarce [4].

The total AGB within a quadrat is calculated by summing the AGB of individual plants. Consequently, the estimation of quadrat-scale AGB serves as a crucial transitional stage in the upscaling process from individual-scale to larger-scale aboveground biomass measurements. AGB at both the individual and quadrat scales can be determined via modeling methods. However, issues such as increased costs and reduced accuracy frequently occur during the process of acquiring large-scale biomass data by using modeling methods. The estimation of quadrat AGB introduces the concept of aboveground biomass density (AGB per unit area, AGB_D_). For larger-scale assessments, AGB can be calculated by multiplying AGB_D_ by the study area, and AGB_D_ can be effectively calculated using modeling methods. Employing this approach not only avoids the conversion of the study area but also circumvents the issue of scale transformation. Although this method has been validated for specific species in desert ecosystems [4,41,42], interactions between different plants can affect the accuracy of AGB estimation in desert areas. Therefore, the applicability of existing AGB_D_ modeling methods to the Gobi Desert urgently requires verification.

To construct AGB models, it is essential to select appropriate morphological susceptibility factors as estimation variables and subsequently identify the optimal fitting model based on evaluation indicators [4,42,43]. Among the various commonly employed fitting models, such as linear, power [44], exponential, and quadratic function models, the linear model can intuitively and efficiently simulate the extent to which variables affect AGB, and the allometric scaling model is the most widely used for estimating plant AGB [45,46,47]. Allometric scaling relationship is a universal phenomenon applicable to all organisms, regardless of individual size, research scale, or species [4]. Additionally, variable selection is the key step in model construction in the desert. The variables basal diameter (BD) and diameter at breast height (DBH) have been widely utilized for estimating biomass in mesic ecosystems like forests [4,48]. The Gobi Desert ecosystem is dominated by multi-stemmed plants like shrubs and herbs, which lack distinct main stems. Moreover, the bases of the plants are always buried by sand, making it challenging to measure BD and DBH. Desert plants are characterized by short stature and large canopy areas. Therefore, plant dimensions reflecting morphological factors such as canopy cover (C) and height (H) are more comprehensive predictors of AGB of desert species due to their better description of desert plant structure [49]. Relative cover (C_r_) has been demonstrated to be applicable in estimating the AGB of specific species across different scales [4,42]. However, the cross-scale applicability of a single-species canopy cover model, which was successfully developed to overcome scale constraints, to multi-species desert communities has yet to be validated.

Based on the selection of fitting variables at the individual and quadrat scales, the applicability of C_r_ in estimating the AGB_D_ of multi-species plant communities in large-scale deserts was examined, and its accuracy, feasibility, and efficiency were verified using both field sampling and remote-sensing data. We hypothesized that (1) canopy-related variables outperform plant height, and allometric models perform better than linear models at individual, quadrat and regional scales; (2) canopy cover can accurately estimate AGB at multiple scales, thereby addressing the scaling issue for multi-species communities. Accordingly, the primary objectives of this study were as follows: (1) to develop estimation models for the AGB of eight dominant species in the Gobi Desert region of Gansu, China, through comparison of different empirical models; (2) to test the performance of the AGB estimation models based on canopy cover at different scales for multi-species desert vegetation. These models will lay a foundation for the quantitative monitoring of AGB and carbon stocks in the Gobi Desert of Gansu Province and similar arid regions worldwide.

## 2. Results

### 2.1. Aboveground Biomass Estimation Models at the Individual Scale

#### 2.1.1. Correlation Between Individual Aboveground Biomass and Morphological Factors

Each of the selected morphological factors was found to exhibit a significant correlation with the individual AGB of all eight plant species (*p* < 0.01, Table 1). This suggests that all the factors, including C_i_, H and C_i_H, can serve as candidate independent variables for establishing AGB estimation models at the individual scale.

For *Ephedra sinica* (*E. sinica*, r = 0.557), *Reaumuria songarica* (*R. songarica*, r = 0.786), *Salsola collina* (*S. collina*, r = 0.692) and *Bassia dasyphylla* (*B. dasyphylla*, r = 0.528), the variable C_i_ exhibited the highest correlation coefficient with AGB, followed by C_i_H. While for the other four species, namely *Nitraria tangutorum* (*N. tangutorum*, r = 0.735), *Alhagi sparsifolia* (*A. sparsifolia*, r = 0.750), *Artemisia desertorum* (*A. desertorum*, r = 0.844) and *Suaeda glauca* (*S. glauca*, r = 0.763), the variable C_i_H showed the highest correlation coefficient with AGB, followed by C_i_. For all species, the variable H exhibited the lowest correlations, indicating that plant height is difficult to utilize for precisely characterizing AGB in desert ecosystems. In contrast, variables related to the canopy projected area (C_i_ & C_i_H) showed strong correlations with AGB.

There was a large discrepancy in sample sizes (e.g., *E. sinica* has n = 46, while *B. dasyphylla* has n = 1392), which was a key determinant of sampling error and exerted a direct influence on the stability of estimation precision. For species with limited sample sizes, the uncertainty associated with their correlation relationships is elevated. Conversely, large sample sizes may produce statistically significant correlations that lack biological relevance. Although the correlation between H and AGB is relatively weak, all three candidate variables were adopted in the construction of estimation models to explore their potential estimation value.

#### 2.1.2. Performance of Aboveground Biomass Estimation Models at the Individual Scale

For each species, three fitting models incorporating different variables were established based on the allometric scaling model. Among these variables, the optimal AGB models for five plant species, specifically *N. tangutorum*, *R. songarica*, *A. sparsifolia*, *S. glauca* and *S. collina*, were established using C_i_H. These models exhibited the highest R^2^ values, along with the lowest RMSE and AICc values, whereas the optimal models for three plant species, namely *E. sinica*, *A. desertorum* and *B. dasyphylla*, were constructed based on C_i_ (Table 2). Generally, most optimal models for plant biomass typically exhibit the lowest values for other indicators when achieving the highest R^2^ values. Although the MAE of plants *A. desertorum* and *B. dasyphylla* did not concur with other evaluation indicators, they were extremely close to the highest values. The TRE values of all AGB estimation models for all species are extremely low, indicating a relatively accurate predictive capacity and no significant deviation in predicted values. Overall, in accordance with correlation analysis, models with factors related to canopy area (C_i_ & C_i_H) as independent variables are suitable for estimating AGB of desert plants, whereas the variable H cannot be used independently for estimating AGB.

**Figure A1:**
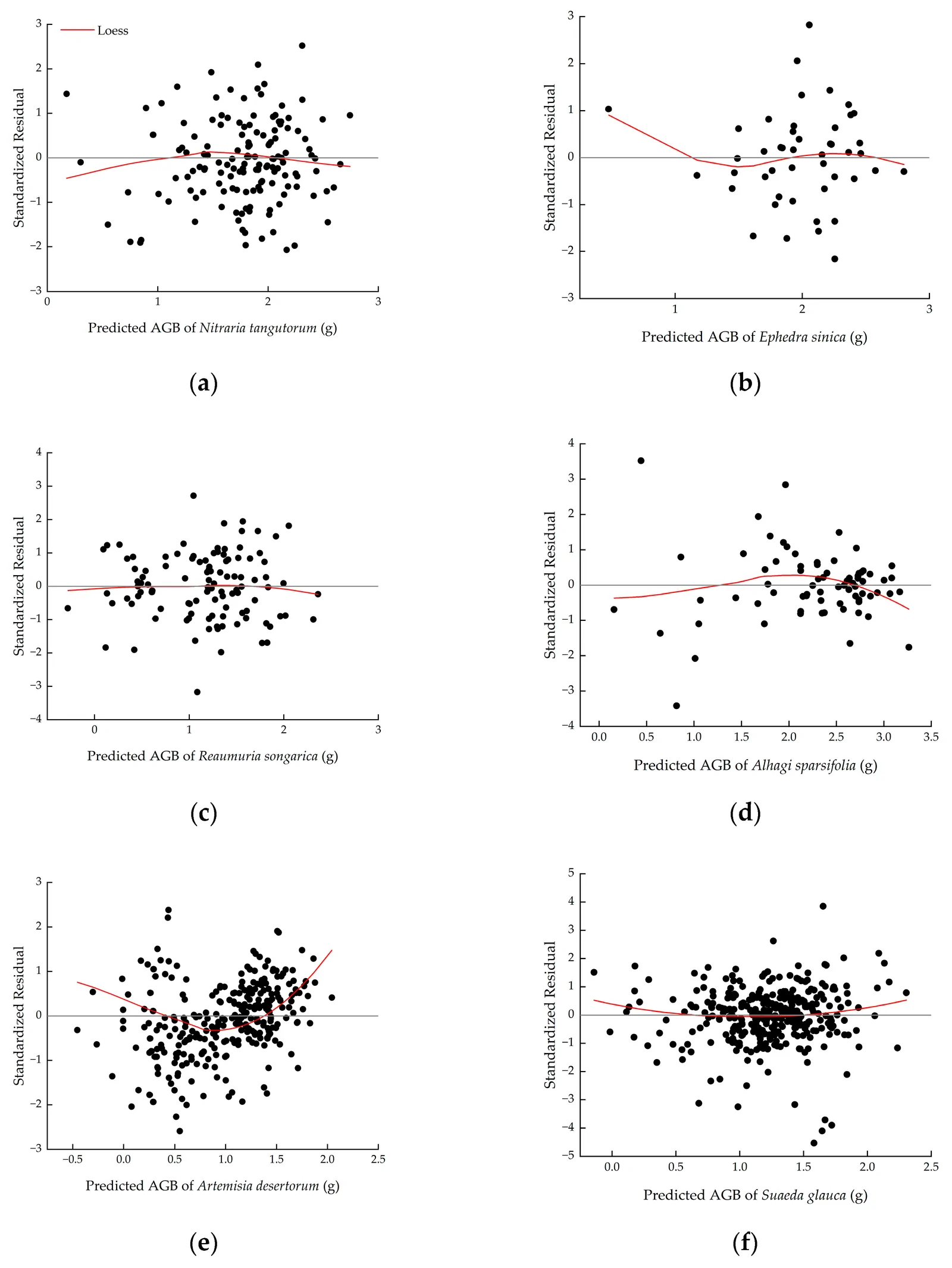

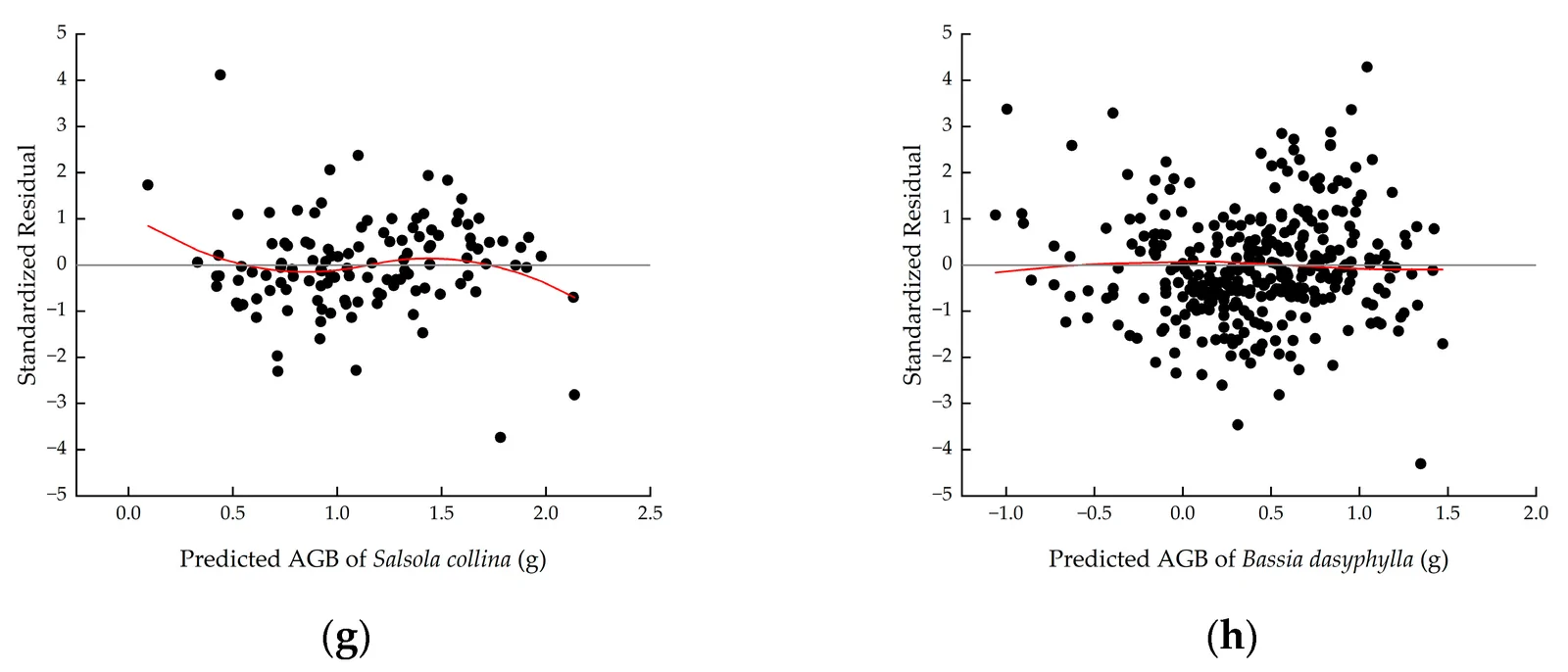
Scatter plot of fitted AGB values and residuals of (**a**) *Nitraria tangutorum*, (**b**) *Ephedra sinica*, (**c**) *Reaumuria songarica*, (**d**) *Alhagi sparsifolia*, (**e**) *Artemisia desertorum*, (**f**) *Suaeda glauca*, (**g**) *Salsola collina*, (**h**) *Bassia dasyphylla*, with superimposed loess local smoothing trend lines (redlines in subfigures).

**Figure A2:**
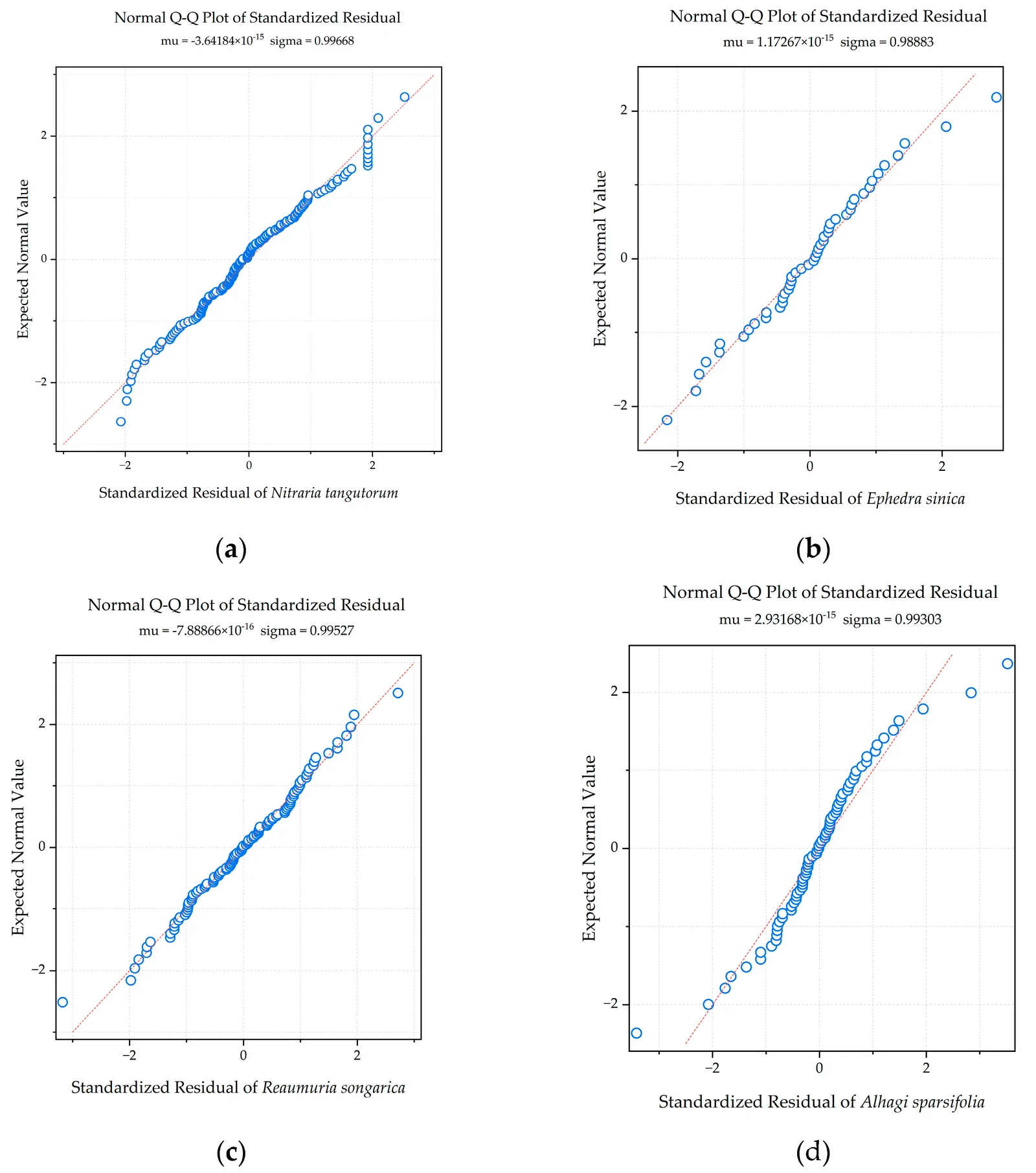

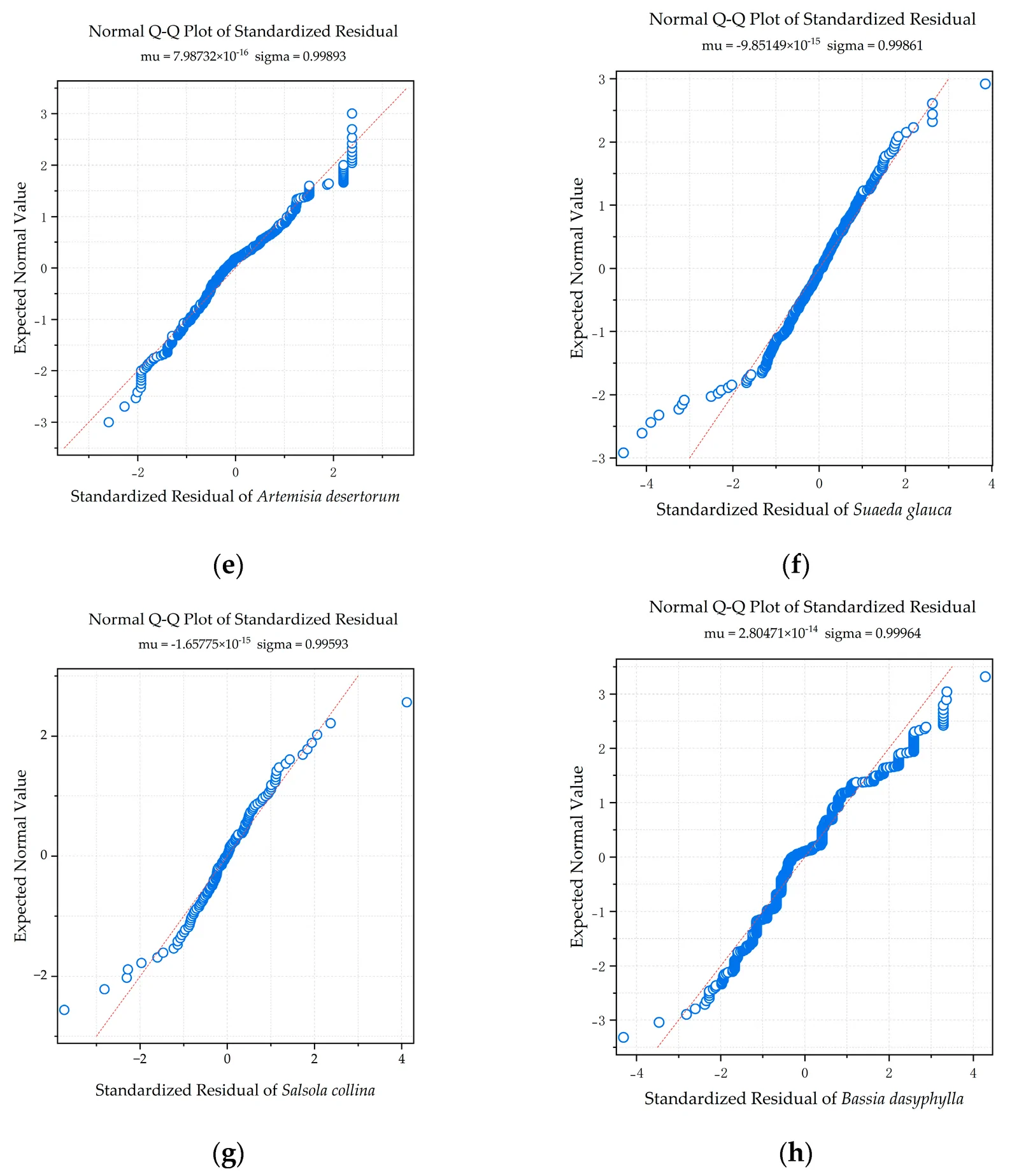
Normal Q-Q plot of standardized residuals of (**a**) *Nitraria tangutorum*, (**b**) *Ephedra sinica*, (**c**) *Reaumuria songarica*, (**d**) *Alhagi sparsifolia*, (**e**) *Artemisia desertorum*, (**f**) *Suaeda glauca*, (**g**) *Salsola collina*, (**h**) *Bassia dasyphylla*. If the scatter points of standardized residuals and expected normal values (blue circles) cluster near the reference line y = x (red dashed lines), the normality assumption for the residuals is tenable.

The Ramsey RESET test was applied to assess the adequacy of the model specification and to determine whether omitted variables were present. The results showed that for *N. tangutorum*, *E. sinica*, *R. songarica*, *A. sparsifolia*, *S. glauca*, and *B. dasyphylla*, the null hypothesis that “the model specification is correct” was not rejected, indicating that the allometric model adequately captures the nonlinear relationship between morphological indicators and biomass and that no systematic functional-form specification bias exists (P_R_ > 0.05, Table 3). In contrast, *A. desertorum* and *S. collina* rejected the null hypothesis (P_R_ < 0.05). Moreover, the Loess line in the residual plot exhibited a pronounced curvature, the tails of the Q–Q plot deviated significantly, estimates for both high and low values showed substantial bias, and the residual normality test was not passed (Figure A1 and Figure A2, Table A1). Appropriate models for the two species, *A. desertorum* and *S. collina*, were explored using various combinations of variables and fitting models, and it was found that a univariate quadratic model provides a suitable model for estimating the AGB of *S. collina* (AGB = −602.279C_i_^2^ + 487.001C_i_ − 1.383, P_R_ = 0.533), whereas no feasible explicit model could be obtained for *A. desertorum*. Overall, the allometric model is a suitable model for estimating the AGB of multi-species desert communities.

### 2.2. Aboveground Biomass Estimation Models at the Quadrat Scale

**Figure 1:**
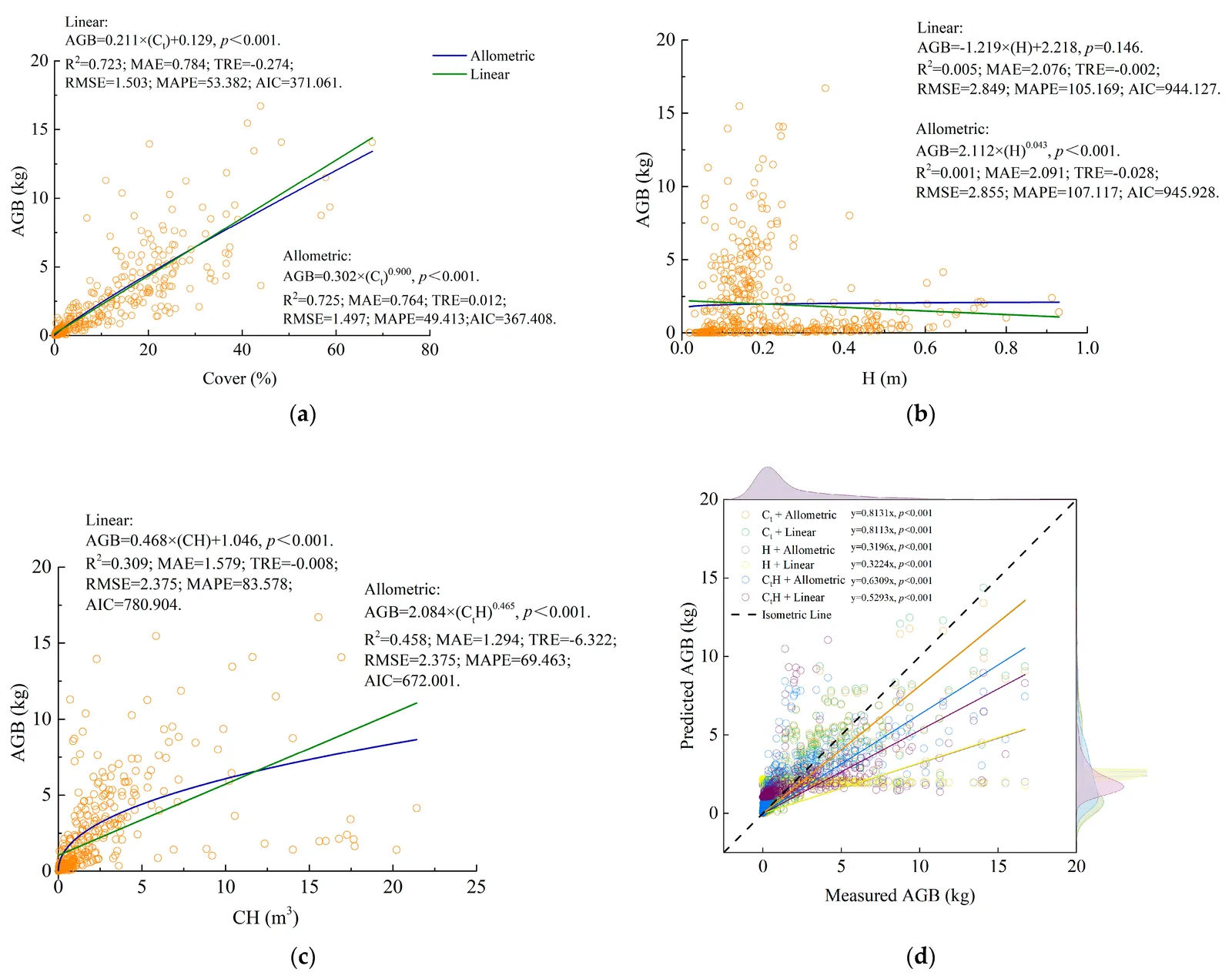
Models of AGB at the quadrat scale. Models fitted by morphological variables, namely: (**a**) total cover (C_t_); (**b**) height (H); and (**c**) canopy volume (C_t_H); (**d**) relationships between the measured and predicted AGB values with different parameters and fitting equations. The black dashed line is isometric with a slope of 1.

Among the three variables, the fitting results of models utilizing C_t_ outperform those of models incorporating the other two variables, H or C_t_H (Figure 1, Table 4). This suggests that C_t_ is a suitable variable for estimating the AGB in the Gobi Desert at the quadrat scale. After determining an appropriate independent variable, the allometric scaling equation was identified through screening, despite the comparable fitting results of allometric and linear functions. Consequently, the optimal AGB estimation model at the quadrat scale was determined as AGB = 0.302 × C_t_^0.900^. This model achieved the highest accuracy (Figure 1a), demonstrated the highest coefficient of determination (R^2^ = 0.725), the lowest error values (RMSE = 1.497), and the lowest AICc value (AICc = 367.408), followed by the fitting result of C_t_H (Figure 1c). In contrast, the model based on H exhibited the weakest predictability (Figure 1b).

Although all models exhibited a significant relationship between measured and predicted AGB (*p* < 0.001) (Figure 1d), the slopes for both C_t_H and H were significantly lower than 1, with a notable deviation in high-value predictions. In contrast, the model based on C_t_ had a slope closest to 1, and the slopes of the allometric and linear models were nearly identical. Consequently, models based on canopy cover can better estimate AGB; however, they also tend to underestimate values.

Cross-validation can effectively mitigate overfitting or underfitting issues that may arise in a model under a specific data partitioning scheme, thereby enhancing the robustness of model evaluation and the model’s generalization capability. At the quadrat scale, 10-fold cross-validation results showed that, whether using allometric or linear models, the AGB predictions based on quadrat cover exhibited high consistency with the measured values; the validation R^2^ and the fitting R^2^ were also very close to each other, demonstrating good model generalization capability (Figure 1d, Table 5). Although the allometric model effectively captured data characteristics within the study area and achieved slightly higher estimation accuracy than the linear model, its stability and generalization capability (R^2^ = 0.7168, RMSE = 1.520, MAE = 0.773) were somewhat weaker than those of the linear model (R^2^ = 0.7187, RMSE = 1.515, MAE = 0.788).

### 2.3. Aboveground Biomass Estimation Models at the Regional Scale

**Figure 2:**
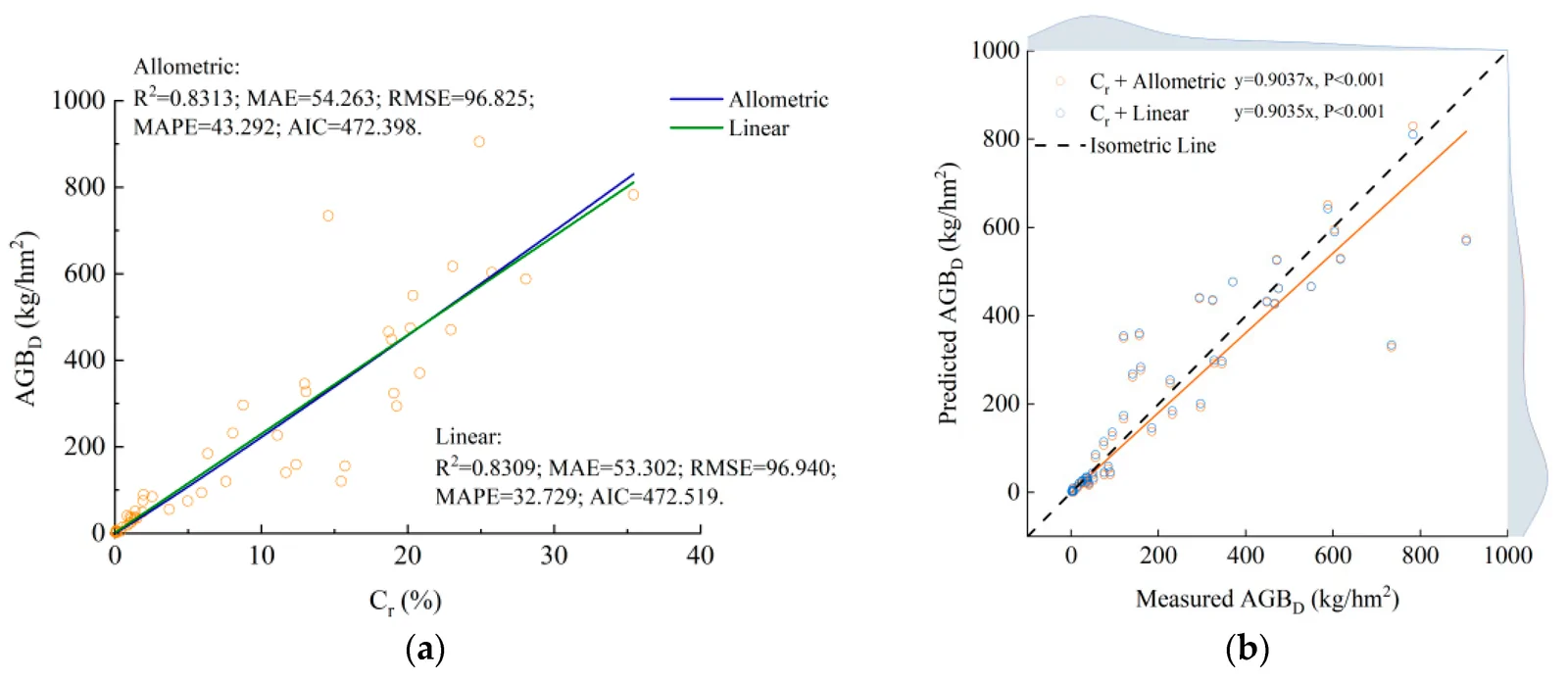
Models of AGB_D_ fitted by field sampling data at the regional scale: (**a**) Models fitted by relative cover (C_r_); (**b**) Relationships between the measured and predicted AGB_D_ values with allometric and linear fitting equations.

In terms of regional-scale AGB estimation, the model based on C_r_ exhibited relatively high R^2^ values and small errors (Figure 2, Table 6). C_r_ was accordingly identified as a suitable variable for estimating the AGB_D_ of desert plants at the regional scale.

Predictive models for AGB_D_ at the regional scale were obtained by fitting linear and allometric functions with C_r_. Similar to the findings at the quadrat scale, the two fitting results were comparable (each R^2^ value was close to 0.831, and the disparities in errors and AIC values were small). Specifically, the allometric model had a slightly higher R2 than the linear model (R^2^ = 0.8313), smaller RMSE (RMSE = 96.825) and AIC (AIC = 472.398) values, yet higher MAE (ΔMAE = 0.961) and TRE (TRE = 1.432) values (Figure 2, Table 6). Consequently, the allometric model was recognized as the most suitable for estimating desert AGB at the regional scale. Based on these evaluations, the optimal Gobi Desert AGB estimation model at the regional scale was determined as follows: AGB = 20.114 × C_t_^1.043^.

At the regional scale, leave-one-plot-out (LOO) cross-validation results showed similar performance to the quadrat scale. Whether using allometric or linear models, AGB predictions based on quadrat cover achieved high consistency with the measured values using both models; the validation R^2^ was close to the fitting R^2^, demonstrating good model generalization capability (Figure 2b, Table 6). Although the allometric model effectively captured data characteristics within the study area and achieved slightly higher estimation accuracy than the linear model, its stability and generalization capability (R^2^ = 0.8087, RMSE =103.1, MAE = 57.92) were somewhat weaker than those of the linear model (R^2^ = 0.8162, RMSE = 101.0, MAE = 55.51).

**Figure 3:**
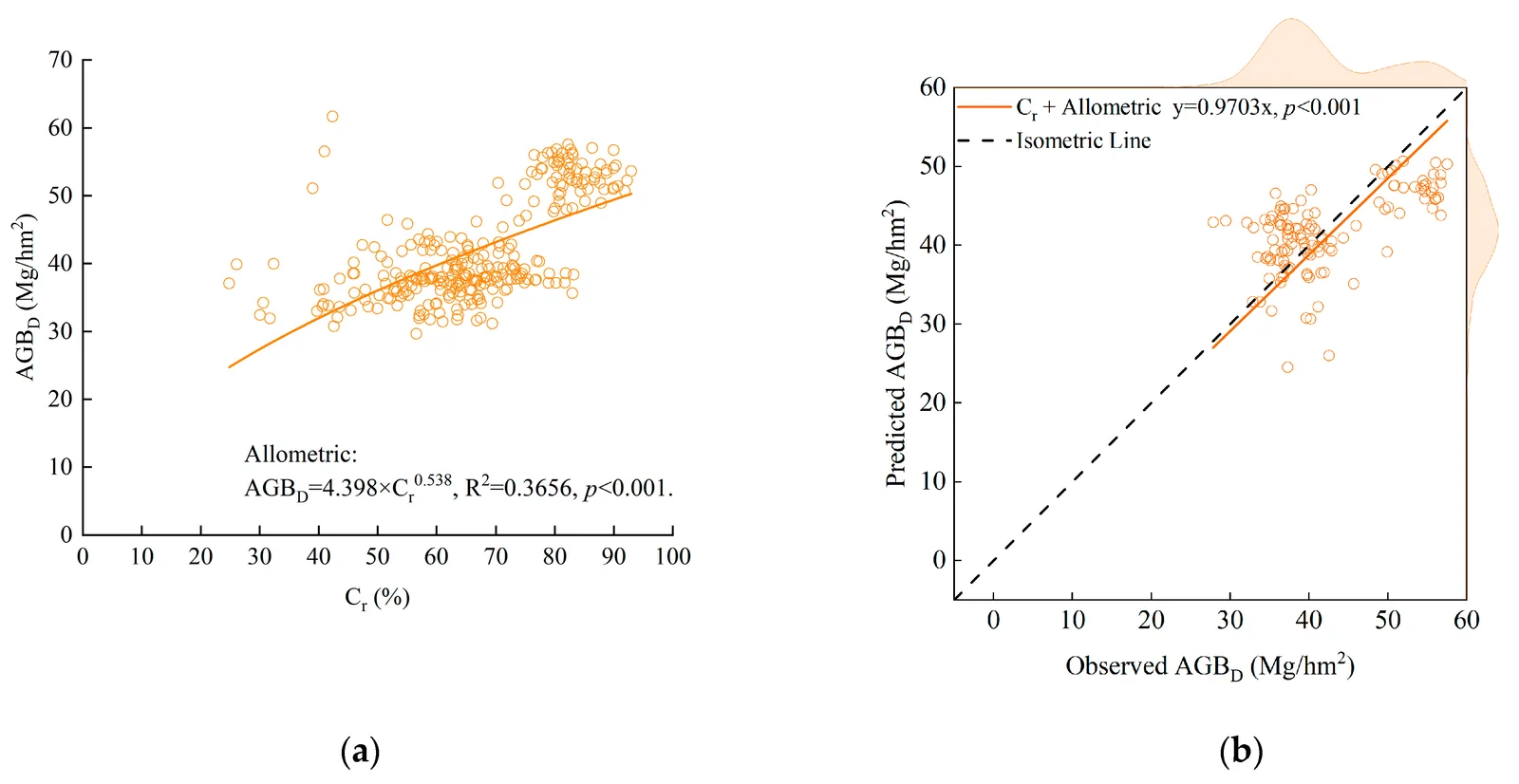
Models of AGB_D_ fitted by remote-sensing data at regional scale: (**a**) Model fitted by relative cover (C_r_); (**b**) Relationships between the observed and predicted AGB_D_ values.

Similar to the fitting results at the quadrat scale, there is a slight difference between the predicted and measured values at the regional scale (Figure 3b), but the C_r_-based estimation model can be employed for accurate estimation of AGB_D_ in arid habitats. In comparison with the model at the quadrat scale, the optimal model at the regional scale exhibits a higher R^2^, lower errors and a lower AIC value, indicating greater accuracy at larger scales.

Compared to field sampling data, the model estimating regional AGB using remote-sensing data exhibits a fitting slope close to 1 (slope = 0.9703 ± 0.0131, Figure 3b), confirming the absence of systematic overestimation or underestimation in biomass predictions and the manageable magnitude of overall model bias. However, the R^2^ value of the AGB model constructed using remote-sensing data is low (R^2^ = 0.3656, Figure 3a). Due to the inherent precision constraints of remote-sensing data, low goodness-of-fit, and high scatter-point dispersion, the model’s ability to explain AGB at the regional scale is limited, and it is exclusively applicable for coarse estimation of community biomass across regional extents.

## 3. Discussion

### 3.1. Aboveground Biomass Estimation Modeling Methods in the Gobi Desert

The establishment of an estimation model using readily measurable factors is a common approach in the research on AGB [50]. Selected factors ought to exhibit a close correlation with the morphology of individual plants. The morphological features of Gobi Desert ecosystems differ from those of mesic ecosystems. As an important morphological parameter, plant height is frequently employed for the estimation of AGB but demonstrates limited predictive capacity in arid habitats [45,49,51]. While canopy area has been proved to be an effective predictor variable for the estimation of AGB [45,52]. Furthermore, for desert plants characterized by short stature and a high branching ratio, using the product of canopy area and plant height (CH), denoted as canopy volume (V), as the independent variable serves as an indicator with strong predictive potential [4].

Nevertheless, plants in the Gobi Desert, predominantly consisting of shrubs and herbs, lack main stems, and the bases of plants are always covered with sand. Using factors such as diameter at breast height (DBH) and basal diameter (BD) [53,54], which are commonly employed to estimate AGB in mesophytic habitats, can easily lead to inaccurate measurements and increase estimation difficulty [55].

In addition, there are some models incorporating multiple variables, such as the model based on D and H [56], the model based on BD, W and H [10,45], and the model based on BD, C and H [40]. These multivariate models are also capable of attaining favorable fitting outcomes, but they are plagued by the issue of overfitting.

Generally, the R^2^ values exhibit an upward trend in tandem with an augmentation in the quantity of variables incorporated into the model. Furthermore, the inclusion of redundant independent variables can give rise to challenges in interpreting regression equations and contribute to an escalation in error [50]. Consequently, with appropriate equation selection, precise estimation results can be acquired by utilizing a limited number of parameters.

In this study, the models incorporating the selected variable C_i_H achieved the optimal fitting results when estimating AGB in the Gobi Desert at the individual scale based on goodness-of-fit and Ramsey RESET test results. Moreover, models based on C_i_ also yielded comparable results. The variables associated with canopy area achieved favorable fitting results, whereas the parameter H only slightly enhanced the fitting results. From the perspectives of accuracy and measurement convenience, it appears that C serves as a simpler and more applicable independent variable for AGB estimation. This proposition has also been verified at both the quadrat (C_t_) and regional (C_r_) scales. Consequently, it is proposed that a more accurate biomass estimation model can be obtained by incorporating multiple variables for screening, with consideration of desert plant morphology, which should be evaluated in further studies on AGB [4].

Upon the determination of candidate independent variables, diverse models, including linear, quadratic, polynomial, and allometric models, were employed for the estimation of AGB [57]. Although linear and polynomial models have achieved favorable fitting results, these empirical models lack mechanistic explanations [58]. It is commonly recognized that allometric growth relationships represent universal laws that are independent of species, individual size, and scale. Moreover, they reflect the synergistic relationships between plant morphological growth and AGB increase [59,60]. The findings of this study offer evidence to support this theory. The widely developed allometric equations for species in mesic climates were also applicable for the estimation of multi-species AGB in arid habitats [54,61], and allometric models were superior to linear models across all studied scales. In this study, fitting results at the quadrat and regional scales were suboptimal (R^2^ values did not exceed 0.850) due to morphological disparities among different species. Future research necessitates more refined model optimization and mechanism elucidation.

### 3.2. Multi-Scale Aboveground Biomass Estimation Methods

The issue of scale transformation has long served as a significant obstacle and focal point in ecological research [4,62] because most ecological processes typically exhibit scale dependence [63]. Numerous studies have been conducted to overcome the limitations of scale transformation and perform large-scale biomass estimation, including the biomass conversion factor method and remote-sensing-based biomass models [64,65]. Nevertheless, the biomass conversion factor method requires comprehensive and detailed inventory data, and remote-sensing methods exhibit lower accuracy in desert regions with sparse vegetation cover [57,66]. Owing to these limitations, these methods are not suitable for estimating the biomass of desert vegetation [4,42].

In research concentrating on specific species like *Alhagi sparsifolia* [41], *Haloxylon ammodendron* [42] and *Asterothamnus centraliasiaticus* [4], AGB in desert regions can be effectively estimated through projected canopy cover. This indicates that plant-projected area alone serves as a valid variable for estimating AGB of desert plants. Moreover, these estimation models, based on allometric equations, have achieved excellent fitting results when estimating the AGB of single species, effectively facilitating scale transformation through plant projection canopy cover. In this study, by constructing optimal estimation models for the AGB of eight dominant desert species within the study area, it was verified that variables related to plant projection area (C_i_ and C_i_H) can effectively estimate the individual AGB of desert plants. When scaled up to the quadrat scale, using the total projected area as the independent variable also enables accurate estimation of AGB. By utilizing C_r_, it is feasible to accurately estimate the AGB_D_ of desert communities at the regional scale, thereby enabling rapid and precise estimation of AGB across desert regions.

The average AGB_D_ across 53 sample plots in the northwest of Gansu province was 204.430 kg/hm^2^, with values ranging from 1.291 to 904.860 kg/hm^2^, indicating strong spatial heterogeneity. Sampling studies conducted in the Junggar Desert in northern Xinjiang revealed an average AGB_D_ of plant communities of 1154.2 kg/hm^2^, with a range of 20–7081.2 kg/hm^2^ [67]. Research in the Gurbantunggut Desert indicated that the average biomass density of three desert communities ranges from 605 to 927 kg/hm^2^, with microhabitats being the core driver of biomass heterogeneity [68]. The primary reason why the AGB_D_ estimates of deserts in the northwest of China conducted at similar latitudes are higher than those in this study is due to higher precipitation and different vegetation. The average AGB_D_ based on remote-sensing data was 41.942 Mg/hm^2^, with a variation range of 27.852–61.674 Mg/hm^2^. The AGB_D_ derived from remote-sensing data is substantially higher than that obtained from field sampling.

Addressing the order-of-magnitude discrepancy between ground-based sampling and remote-sensing inversion of biomass density, we conducted a quantitative decomposition and source analysis across five dimensions: statistical scope, mixed-pixel effects, inversion errors in remote-sensing data, differences in modeling methods, and spatiotemporal matching, with a particular focus on clarifying the contributions of mixed-pixel effects caused by spatial resolution and the systematic errors inherent in the remote-sensing data sources themselves. Using a logarithmic decomposition method to quantitatively analyze the sources of the biomass order-of-magnitude discrepancy, the results showed that mixed-pixel effects caused by spatial resolution are the primary contributing factor, accounting for 35.67% of the total discrepancy. Differences in statistical scale and systematic errors in remote-sensing data sources account for 23.96% and 22.51%, respectively. Moreover, contributions of modeling factors and spatiotemporal matching account for 10.93% and 6.93%, respectively, which are relatively small. As the automated vegetation monitoring system, comprising high-resolution satellite networks, low-altitude UAV remote sensing, and AI-based intelligent interpretation, continues to improve, mixed-pixel effects and data-source systematic errors can be effectively mitigated. Consequently, the accuracy of large-scale biomass estimation and the level of operational monitoring in arid regions are expected to continue improving in the future [69].

Spatial scale mismatch is a common cause of numerical discrepancies between ground sampling and remote-sensing inversion results, a phenomenon that is particularly pronounced in arid desert ecosystems characterized by strong heterogeneity. Existing studies have shown that scale differences can lead to relative estimation errors exceeding 30%, making them one of the primary sources of uncertainty in ecological remote-sensing estimates [70], which is consistent with the findings of this study. To address the systematic bias introduced by scale differences, a multi-scale assessment and calibration framework can be adopted: using 400 m as an intermediate reference scale to perform scale conversion and result calibration between ground quadrats and satellite pixels, thereby effectively reducing estimation errors caused by spatial mismatch [70]. As high-resolution satellite networks expand and automated vegetation monitoring systems continue to improve, the effects of mixed-pixel phenomena and systematic errors in data sources can be further mitigated, which is expected to continuously enhance the accuracy and stability of large-scale biomass estimates in arid regions.

We developed a method for estimating AGB of desert plant communities without scale limitations. This modeling method possesses high utility value. It estimates the regional AGB_D_ based on the readily measurable factor C_r_ and then combines it with the regional area to obtain the total AGB. While enabling accurate and rapid estimation, it significantly simplifies the process of AGB estimation and conserves the resources of labor and material. This method not only overcomes scale limitations but, as shown by the study’s results, exhibits a tendency of improved model fit with increasing scales. There are two primary reasons for the improved goodness-of-fit at the regional scale. First, in the Gobi Desert, where vegetation is sparse and distributed in highly patchy patterns, increasing the sample plot area reduces the influence of extreme values and enhances the stability of the relationship between vegetation cover and AGB. Second, when quadrats containing substantial random fluctuations are merged, the independent random errors are smoothed out through averaging in the larger sample plots.

In the future, integrating multi-source data on desert C_r_ obtained through unmanned aerial vehicle photography and remote-sensing technologies can further enhance the accuracy and efficiency of AGB estimation in desert regions.

The applicability of the cover allometric model for estimating AGB in multi-species communities rests on the accurate estimation of AGB of individual desert plants by the allometric model. Accordingly, in Gobi Desert regions similar to the study area, the cover allometric model exhibits strong applicability, enabling relatively accurate biomass estimates and facilitating the development of a general model. Cross-validation results also support this conclusion. Nonetheless, although the spatial scope of this study is broad, the extreme habitat conditions across study sites are relatively homogeneous. Within the study area, plant species for which the allometric model fails to fully explain AGB variability (e.g., *A. desertorum* and *S. collina*) may reduce model accuracy. If such plant species are more prevalent in other desert regions, this may further exacerbate model instability and diminish its predictive capacity. Furthermore, in habitats with markedly different environmental conditions, variations in plant morphology may further reduce the accuracy of models relying on the single variable. Overall, the cover allometric model demonstrates strong applicability for estimating AGB in Gobi Desert regions of northwestern China, allowing for convenient acquisition of sufficiently accurate biomass estimates. However, the applicability of this model in other arid regions warrants further investigation and validation.

## 4. Materials and Methods

### 4.1. Study Area

**Figure 4:**
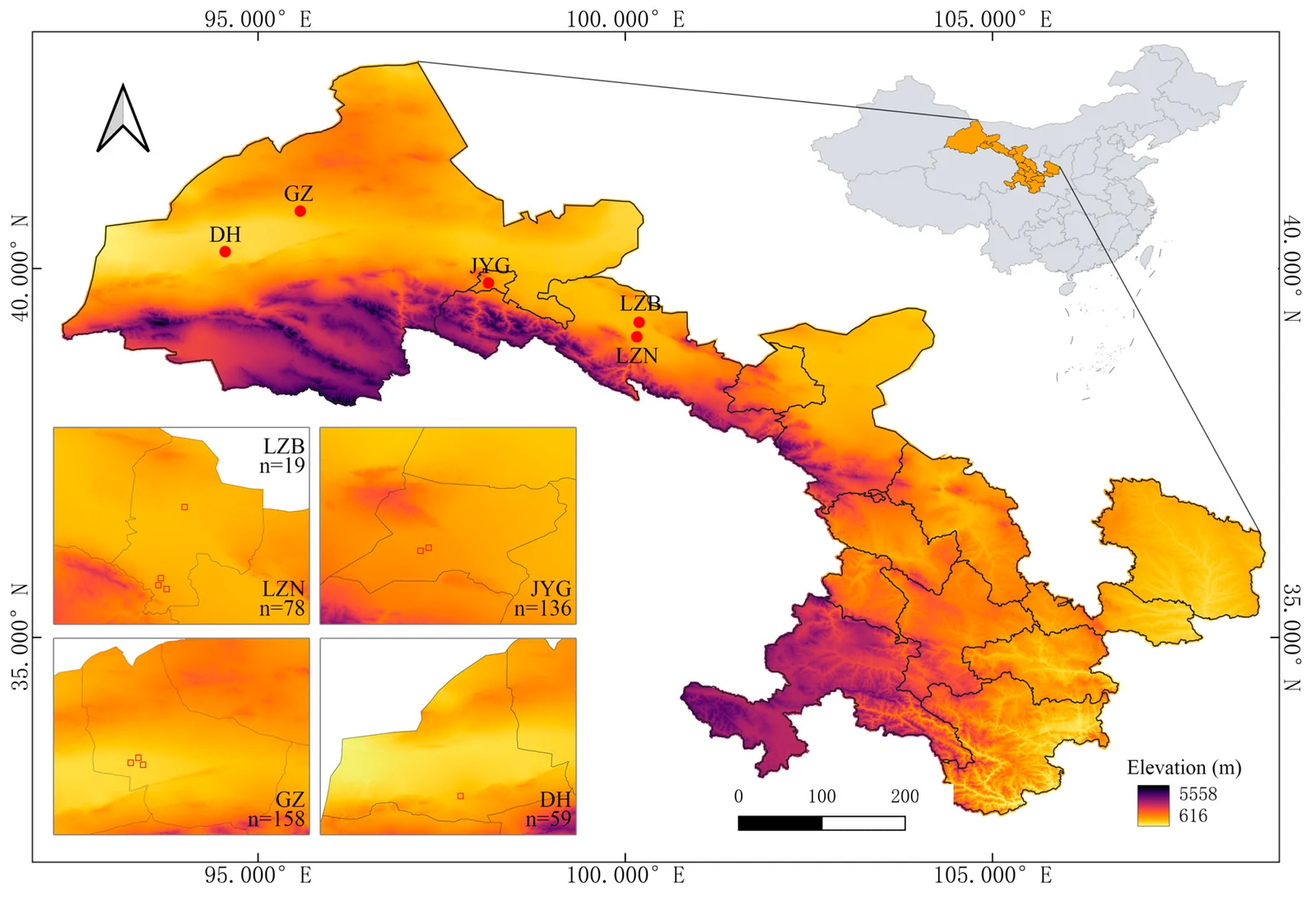
Locations of the 5 study sites and the altitude in Gansu Province. The n values in the subplots are the number of sampling quadrats.

The study area is located in the northwestern part of Gansu Province, China (94°18′–100°7′ E, 39°3′–40°41′ N, Figure 4). This region is characterized by a temperate continental climate, with annual precipitation decreasing from 170 mm in the southeast to 40 mm in the northwest, and approximately 60% of the precipitation is concentrated from June to August [16]. The annual average temperature spans from 5 to 12 °C, gradually decreasing from the southeast to the northwest. The climatic conditions exhibit a smooth gradient transition from the southeast towards the northwest. The main soil types in the study area are similar, including brown desert soils and gray-brown desert soils, with an average gravel coverage of 52% [71]. The vegetation in this region is dominated by drought-tolerant plants, such as *Nitraria tangutorum*, *Reaumuria songarica*, *Suaeda glauca*, *Salsola collina*, *Bassia dasyphylla*, *Alhagi sparsifolia*, *Artemisia desertorum*, and *Ephedra sinica*. In light of the geographical and climatic features, it is an ideal region for the construction and verification of AGB estimation models.

### 4.2. Sampling and Measurements

From 2015 to 2017, field surveys were conducted in Linze (LZB & LZN), Jiayuguan (JYG), Guazhou (GZ) and Dunhuang (DH) during the period of vigorous plant growth from July to August for three consecutive years. All sampling quadrats were 10 m × 10 m in size and were at a sufficient distance from transmission lines, roads, structures, and ditches to reduce possible disturbances. Following the random selection of a starting point, 10 sample plots were surveyed continuously along a straight line. Where sufficient single-row sample plots could not be obtained due to topographic or other constraints, a parallel two-row arrangement with 5 sample plots per row was employed. All plant individuals in the quadrats were measured. For each plant individual, the height (H) and crown width (W_1_ × W_2_) were recorded.

A total of 450 sampling quadrats were established in 5 sites, and 2944 individual plant samples were collected. For herbs in the quadrat, the entire aboveground part of each individual was collected. For shrubs and subshrubs, the partial harvest method was employed to reduce damage to the habitat [16]. The horizontal projection of plant canopy was approximated as a circle or an ellipse, which was then divided along the radial direction into 8 fan-shaped sampling units of equal area. A set of opposite fan-shaped units, totaling 1/4 of the total canopy projected area, was selected, and all aboveground tissues within this area were harvested, and their dry matter mass was measured as a 1/4 plant AGB, from which the total AGB of the entire plant was calculated. This symmetrical sampling design can effectively offset estimation bias caused by canopy orientation heterogeneity and uneven distribution of plant biomass while reducing vegetation damage.

Following collection, plant samples were air-dried to eliminate weighing errors resulting from mold growth or tissue decay during transportation. Prior to oven drying, the samples were cut into small fragments to facilitate efficient processing of large sample batches. Subsequently, each plant sample was oven-dried at 70 °C in the laboratory until a constant weight was achieved, after which the dry weight was measured.

### 4.3. Modeling of Multi-Scale Biomass Estimation

The allometric scaling model (1) was adopted for AGB estimation of all species at the individual scale. While at the quadrat and regional scales, linear (2) and allometric scaling models were fitted separately, and the resulting functions were compared to determine the optimal biomass estimation model. The allometric scaling equation and linear equation can be expressed as follows:

(1) $$\mathrm{AGB}=\beta_{i}\times X_{i}^{\alpha_{i}}$$

AGB = a_i_X_i_ + b_i_ (2)

where AGB is aboveground biomass, β_i_ is a normalization constant, X_i_ is a certain vegetation morphological characteristic (e.g., cover, height), and α_i_ is the allometric exponent; a_i_ and b_i_ are the required constant coefficients.

#### 4.3.1. Individual Scale Modeling

To obtain the optimal rules for modeling the AGB of plants in arid regions, morphological data of a total of 2725 individuals belonging to 8 dominant species, namely *Nitraria tangutorum*, *Ephedra sinica*, *Reaumuria songarica*, *Alhagi sparsifolia*, *Artemisia desertorum*, *Suaeda glauca*, *Salsola collina* and *Bassia dasyphylla*, were collected to establish desert AGB estimation models separately at the individual scale. The collected plant samples encompass almost all plant types found in the Gobi Desert. Among them, *N. tangutorum* and *E. sinica* were perennial shrubs, *R. songarica* and *A. sparsifolia* were perennial subshrubs, *A. desertorum* was a perennial herb, while *S. glauca*, *S. collina* and *B. dasyphylla* were annual herbs. The number of 8 dominant species accounts for 92.49% of all sampled individuals, while their projected area accounts for 96.50% of the total projected area of vegetation, which can represent the AGB accumulation status of vegetation in the study area. The remaining 219 plant individuals belong to 11 species. Among these species, *Atraphaxis pungens*, *Haloxylon ammodendron*, *Kalidium foliatum*, *Caragana korshinskii* and *Calligonum mongolicum* were perennial shrubs; *Caroxylon passerinum* and *Sympegma regelii* were perennial subshrubs; *Peganum harmala* and *Artemisia frigida* were perennial herbs; *Agriophyllum pungens* and *Halogeton glomeratus* were annual herbs. Due to the relatively small sample sizes, no individual AGB estimation models were developed for these species.

Numerous studies have been conducted on the establishment of models for estimating AGB in arid regions. Based on previous research, three parameters, including Ci (individual canopy area), H and their composite factor C_i_H, were selected to fit the AGB of eight dominant desert plants using the allometric equation [4,42]. Assuming the projection of a plant crown to be an ellipse, the projected area of an individual plant can be calculated based on the crown widths using the ellipse area formula. The factor C_i_H serves as a simulation of plant canopy volumes, simulating a cylinder or a cone. The equations used to derive C_i_ and C_i_H are as follows:

(3) $$C_{i}=\pi \times \frac{W_{1}}{2}\times \frac{W_{2}}{2}$$

C_i_H = C_i_ × H (4)

where Ci is the canopy area of an individual plant (m^2^); W_1_ and W_2_ are crown widths (m); C_i_H is canopy volume (m^3^); H is the plant height (m).

#### 4.3.2. Quadrat Scale Modeling

At the quadrat scale, a more comprehensive survey of the morphological factors of all individual plants (not only the 8 dominant species) was conducted within 450 quadrats. The quadrat AGB was calculated by summing the biomasses of all individual plants in the quadrats. However, this method is impractical for large quadrats due to its overly cumbersome calculations. Some studies have shown that canopy cover is a more appropriate parameter for estimating desert plant AGB over larger areas [4,42]. Therefore, we attempted to estimate AGB by using the total canopy cover (C_t_, m^2^) of all plant species in the sample plot. In this study, 3 variables, including C_t_, H and C_t_H, were selected to fit AGB using the allometric equation at the quadrat scale. The composite factor C_t_H denotes the volume occupied by the plant community in the quadrat. The equations used to derive C_t_ and C_t_H are as follows:

(5) $$C_{t}=\sum C_{i}$$

C_t_H = C_t_ × H (6)

where C_t_ is plant total canopy cover, C_t_H is quadrat canopy volume (m^3^); H denotes the mean plant height in the quadrat.

#### 4.3.3. Regional Scale Modeling

Although C_t_ has demonstrated substantial applicability in estimating AGB at the quadrat scale, it cannot be directly applied at larger scales. Conducting comprehensive determinations of C_t_ at larger scales is unfeasible. Even if C_t_ is determined, it would be necessary to convert area estimates to a fixed sample area to utilize the model for biomass estimation [4,42]. Furthermore, these values must be multiplied by the area multiple to obtain the values for the total AGB [4].

The concept of biomass density has been proposed and verified in the single-species biomass estimation of *Haloxylon ammodendron* [42] and *Asterothamnus centraliasiaticus* [4]. In this study, an attempt was made to estimate the aboveground biomass density (AGB_D_, kg/hm^2^) of desert plant communities at the regional scale by utilizing relative canopy cover (C_r_, %), which refers to the total cover of desert vegetation expressed as a percentage of the total plot area. Ten spatially contiguous 10 m × 10 m quadrats mentioned above were combined into a larger sample plot (0.1 hm^2^, either arranged in 2 rows of 5 quadrats each or 10 quadrats in a single row). For sample plots with insufficient area, regional AGB_D_ is calculated based on the measured actual biomass and actual area. Furthermore, remote-sensing datasets encompassing vegetation cover [72] and aboveground biomass [73] data were adopted to validate the AGB estimation model applicable to broader regional scales. Specifically, the vegetation cover dataset “China regional 250 m fractional vegetation cover data set (2000–2024)” was monthly fractional vegetation cover in China from 2000 to 2024, with a spatial resolution of 250 m; the AGB dataset “A 30-m aboveground biomass dataset for China in 2020” was a nationwide dataset of aboveground biomass density (AGBD) for China in 2020, with a high-resolution of 30 m, which integrates multiple vegetation types. The resolution of both datasets was standardized to 250 m prior to analysis. Employing C_r_ for calculation can be applied to biomass estimation in areas of varying sizes. This approach not only avoids the conversion of the study area as described above but also circumvents the issue of scale transformation [4].

C_r_ = C_t_/A (7)

where C_r_ is relative cover (%), C_t_ is plant total canopy cover; A denotes the area of each quadrat (m^2^).

### 4.4. Model Evaluation

Several indices were used to evaluate the goodness of fit of the models at all scales: coefficient of determination (R^2^), mean absolute error (MAE), total relative error (TRE), root mean square error (RMSE) and Akaike information criterion (AICc). The formulas are as follows:

(8) $$R^{2}=1-\sum_{i=1}^{n}{\left(y_{i}-{\hat{y}}_{i}\right)}^{2}/\sum_{i=1}^{n}{\left(y_{i}-{\bar{y}}_{i}\right)}^{2}$$

(9) $$\mathrm{MAE}=\frac{1}{n}\sum_{i=1}^{n}\left|y_{i}-{\hat{y}}_{i}\right|$$

(10) $$\mathrm{TRE}=\sum_{i=1}^{n}\left(y_{i}-{\hat{y}}_{i}\right)/\sum_{i=1}^{n}y_{i}\times 100\%$$

(11) $$\mathrm{RMSE}=\sqrt{\frac{1}{n}\sum_{i=1}^{n}{\left(y_{i}-{\hat{y}}_{i}\right)}^{2}}$$

where n is the sample size, y_i_ is the measured AGB, ŷ_i_ is the estimated AGB, and ӯ_i_ is the average of measured values.

(12) $$\mathrm{AICc}=\log\frac{\mathrm{RSS}}{n}+2k+\frac{2k(k+1)}{n-k-1}$$

ΔAICc = AICc_i_ − AICc_min_, for i = 1, 2, … (13)

where AICc is the Akaike information criterion, and ΔAICc is the AICc difference; k is the number of parameters. The larger the R^2^ value and the smaller the values of other indices, the smaller the difference between the estimated and measured values of the model, which indicates a better estimation effect of the model.

### 4.5. Predictive Performance

Predictive performance was obtained by cross-validation. In this study, one scheme matching the sampling structure of each scale was adopted. At the quadrat scale, where the sample was large (n = 450) and collected at a fixed set of sites and years, random 10-fold cross-validation was used, which estimates the interpolation accuracy of the model for new quadrats drawn from the same sampling population. Specifically, all 450 quadrats were randomly assigned to 10 folds of 45 (random seed 42). In each of the 10 iterations, the model was calibrated on nine folds and used to predict the remaining fold, so that every quadrat was predicted exactly once. At the regional scale, where the sample was small (n = 53), leave-one-plot-out (LOO) cross-validation was used, which estimates the accuracy of the model for an unsurveyed plot within the sampled region. Each of the 53 plots was withheld in turn and predicted from a model calibrated on the other 52, giving 53 folds. In both schemes, every observation is predicted exactly once, and all predictive statistics are computed on the pooled out-of-fold predictions.

### 4.6. Statistical Analysis

Correlation analysis was conducted in SPSS 20. The AGB at all scales were estimated in Origin 9.0 software. At the individual scale, only power functions were fitted, whereas at the quadrat and regional scales, linear and allometric scaling equations were used. All data were collated using Microsoft Excel 2010; model fitting and plotting were performed using Origin 9.0. Linear relationships were estimated using ordinary least squares (OLS) regression, while nonlinear fitting was performed using the Levenberg–Marquardt (L-M) method. Cross-validation and the Ramsey RESET test were conducted using R 4.2.3.

## 5. Conclusions

In this study, we proposed a multi-scale AGB estimation method for Gobi Desert plants based on canopy cover. Following rigorous validation of its accuracy and effectiveness in estimating AGB of dominant species at the individual level, this method was further extended to multi-species communities at larger scales.

Results indicated that the allometric model was verified to be applicable at the individual, quadrat and regional scales. In terms of variable selection, variables associated with canopy cover were identified as appropriate variables for estimating the AGB of dominant species at the individual scale. At the quadrat scale, C_t_ emerged as the most suitable parameter for estimating the AGB of plant communities. At the regional scale, C_r_ was recognized as optimal for estimating AGB_D_. By integrating C_r_ with the regional area estimates, AGB_D_ can be precisely quantified at any desired spatial scale. This finding is of substantial significance for the management, development, and vegetation research of desert ecosystems.

AGB estimation models based on canopy cover not only overcome scale limitations but, as shown by the study’s results, exhibit a tendency of improved model fit with increasing scales. However, when applied to remote-sensing data, the model exhibits relatively low fitting accuracy. It demonstrates good stability when applied to Gobi Desert habitats similar to the study area, but its applicability in other arid regions still requires further validation.

## Figures and Tables

**Table 1:** Correlations between individual plant AGB and morphological factors, including canopy area (C_i_), height (H) and canopy volume (C_i_H).

Pearson’s r	n	C_i_	H	C_i_H
*N. tangutorum* AGB	152	0.734 ** ^1^	0.419 **	0.735 ** ^2^
*E. sinica* AGB	46	0.557 **	0.298 *	0.463 **
*R. songarica* AGB	107	0.786 **	0.690 **	0.757 **
*A. sparsifolia* AGB	73	0.622 **	0.666 **	0.750 **
*A. desertorum* AGB	469	0.834 **	0.688 **	0.844 **
*S. glauca* AGB	362	0.694 **	0.633 **	0.763 **
*S. collina* AGB	124	0.692 **	0.608 **	0.635 **
*B. dasyphylla* AGB	1392	0.528 **	0.355 **	0.462 **

^1^ *: *p* < 0.05; **: *p* < 0.01. ^2^ Highest Pearson’s r value for each species highlighted in bold.

**Table 2:** Fitting equations for AGB of eight dominant desert plants at the individual scale.

Species	Fitting Equations	R^2^	MAE	TRE	RMSE	ΔAIC	*p*
*N. tangutorum*	AGB = 152.504 × C_i_^0.843^	0.708	0.218	0.000	0.281	14.777	<0.001
	AGB = 763.713 × H^1.864^	0.469	0.303	0.000	0.378	104.449	<0.001
	**AGB = 317.922 × C_i_H^0.674^** ^1^	**0.735**	0.212	0.000	**0.267**	**0.000**	<0.001
*E. sinica*	**AGB = 289.734 × C_i_^0.887^**	**0.623**	0.238	0.000	**0.312**	**0.000**	<0.001
	AGB = 410.554 × H^0.807^	0.136	0.384	0.000	0.472	36.524	0.012
	AGB = 604.811 × C_i_H^0.607^	0.528	0.267	0.000	0.349	9.887	<0.001
*R. songarica*	AGB = 230.330 × C_i_^1.095^	0.836	0.188	−0.001	0.235	8.534	<0.001
	AGB = 819.710 × H^2.302^	0.611	0.286	−0.002	0.361	98.901	<0.001
	**AGB = 493.469 × C_i_H^0.825^**	**0.848**	0.181	0.000	**0.226**	**0.000**	<0.001
*A. sparsifolia*	AGB = 243.254 × C_i_^1.329^	0.755	0.125	0.000	0.373	10.606	<0.001
	AGB = 2527.842 × H^2.522^	0.538	0.169	0.000	0.511	55.589	<0.001
	**AGB = 655.209 × C_i_H^1.010^**	**0.789**	0.115	0.000	**0.346**	**0.000**	<0.001
*A. desertorum*	**AGB = 141.241 × C_i_^0.861^**	**0.631**	0.295	0.001	**0.361**	**0.000**	<0.001
	AGB = 307.440 × H^1.739^	0.440	0.358	0.000	0.444	194.841	<0.001
	AGB = 249.281 × C_i_H^0.629^	0.621	0.293	0.000	0.366	13.059	<0.001
*S. glauca*	AGB = 149.779 × C_i_^0.939^	0.680	0.185	0.000	0.277	14.777	<0.001
	AGB = 576.169 × H^2.093^	0.544	0.257	0.000	0.330	104.449	<0.001
	**AGB = 313.538 × C_i_H^0.726^**	**0.714**	0.179	0.001	**0.262**	**0.000**	<0.001
*S. collina*	AGB = 207.176 × C_i_^0.841^	0.710	0.203	0.000	0.273	9.209	<0.001
	AGB = 860.895 × H^1.973^	0.523	0.266	−0.001	0.350	70.105	<0.001
	**AGB = 462.253 × C_i_H^0.659^**	**0.731**	0.187	−0.001	**0.263**	**0.000**	<0.001
*B. dasyphylla*	**AGB = 176.946 × C_i_^0.994^**	**0.772**	0.233	−0.003	**0.292**	**0.000**	<0.001
	AGB = 87.303 × H^1.614^	0.250	0.457	−0.006	0.530	1657.720	<0.001
	AGB = 683.345 × C_i_H^0.809^	0.754	0.226	−0.010	0.303	107.955	<0.001

^1^ Optimal models with the highest R^2^ and the lowest RMSE and AICc values highlighted in bold.

**Table 3:** Ramsey RESET test for optimal biomass estimation models based on allometric models for eight plant species.

Species	F	df1	df2	P_R_
*N. tangutorum*	0.314	2	148	0.731
*E. sinica*	1.019	2	42	0.370
*R. songarica*	0.178	2	103	0.837
*A. sparsifolia*	2.024	2	69	0.140
*A. desertorum*	12.652	2	465	<0.050
*S. glauca*	1.633	2	358	0.197
*S. collina*	7.302	2	120	<0.050
*B. dasyphylla*	0.711	2	1388	0.491

**Table 4:** Fitting equations for AGB of desert communities at the quadrat scale.

Fitting Equations	R^2^	ΔMAE	TRE	ΔRMSE	ΔAIC	*p*
**AGB = 0.302 × C_t_^0.900^** ^1^	**0.725**	0.000	−0.274	**0.000**	**0.000**	<0.001
AGB = 0.211 × C_t_ + 0.129	0.723	0.020	0.012	0.006	3.653	<0.001
AGB = 2.112 × H^0.043^	0.001	1.327	−0.028	1.358	578.520	<0.001
AGB = −1.219 × H+2.218	0.005	1.313	−0.002	1.352	576.719	0.146
AGB = 2.084 × C_t_H^0.465^	0.458	0.531	−6.322	0.606	304.593	<0.001
AGB = 0.468 × C_t_H + 1.046	0.309	0.815	−0.008	0.878	413.496	<0.001

^1^ Optimal model with the highest R^2^ and the lowest RMSE and AICc values highlighted in bold.

**Table 5:** Out-of-fold comparison of the allometric (A) and linear (L) model forms under identical fold partitions.

Scale	Validation Scheme	R^2^ (A)	R^2^ (L)	RMSE (A)	RMSE (L)	MAE (A)	MAE (L)
Quadrat (n = 450)	Full data fitting (reference)	0.7253	0.7230	1.497	1.503	0.764	0.784
	10-fold CV, random	0.7168	0.7187	1.520	1.515	0.773	0.788
Regional (n = 53)	Full data fitting (reference)	0.8313	0.8309	96.83	96.94	54.26	53.30
	Leave-one-plot-out (LOO)	0.8087	0.8162	103.1	101.0	57.92	55.51

**Table 6:** Fitting equations for AGB_D_ of desert plants based on relative cover (C_r_) at the regional scale.

Fitting Equations	R^2^	ΔMAE	TRE	ΔRMSE	ΔAIC	*p*
**AGB_D_ = 20.114 × C_r_^1.043^** ^1^	**0.** **8313**	0.961	1.432	**0.000**	**0.000**	<0.001
AGB_D_ = 22.859 × C_r_ + 1.094	0.8309	0.000	0.000	0.115	0.121	<0.001

^1^ Optimal model with the highest R^2^ and the lowest RMSE and AICc values highlighted in bold.

## Data Availability

The original data presented in the study are openly available in FigShare at http://doi.org/10.6084/m9.figshare.33170756.v1.

## Abbreviations

The following abbreviations are used in this manuscript:

AGB	Aboveground biomass
AGB_D_	Aboveground biomass density
C_i_	Individual canopy cover at the individual scale
C_t_	Total canopy cover at the quadrat scale
C_r_	Relative canopy cover at the regional scale
H	Plant height
CH	Canopy volume

## Appendix A

**Table A1:** Shapiro–Wilk normality test results.

Species	W	df	*p*
*N. tangutorum*	0.985	152	0.106
*E. sinica*	0.983	46	0.738
*R. songarica*	0.993	107	0.861
*A. sparsifolia*	0.937	73	0.001
*A. desertorum*	0.979	469	<0.050
*S. glauca*	0.949	361	<0.050
*S. collina*	0.952	124	<0.050
*B. dasyphylla*	0.958	1392	<0.050

## References

[1] Kindermann L., Dobler M., Niedeggen D., Linstädter A. (2022). A new protocol for estimation of woody aboveground biomass in disturbance-prone ecosystems. Ecol. Indic..

[2] Xu M., Zhang S., Wen J., Yang X. (2019). Multiscale spatial patterns of species diversity and biomass together with their correlations along geographical gradients in subalpine meadows. PLoS ONE.

[3] Peng S., He N., Yu G., Wang Q. (2017). Aboveground biomass estimation at different scales for subtropical forests in China. Bot. Stud..

[4] Li Z., Ma Y., Wang Y., Liu Y. (2023). Multi-scale biomass estimation of an alpine desert shrub based on relative cover: A case study of Asterothamnus centraliasiaticus in the Qaidam Desert on the Qinghai–Tibet Plateau. Ecol. Inform..

[5] Návar J., Ríos-Saucedo J., Pérez-Verdín G., de Jesús Rodríguez-Flores F., Domínguez-Calleros P.A. (2013). Regional aboveground biomass equations for North American arid and semi-arid forests. J. Arid. Environ..

[6] Poulter B., Frank D., Ciais P., Myneni R.B., Andela N., Bi J., Broquet G., Canadell J.G., Chevallier F., Liu Y.Y. (2014). Contribution of semi-arid ecosystems to interannual variability of the global carbon cycle. Nature.

[7] Ma Y., Im J., Zhen Z., Zhao Y. (2025). A framework for upscaling aboveground biomass from an individual tree to landscape level and qualifying the multiscale spatial uncertainties for secondary forests. Geo-Spat. Inf. Sci..

[8] Qian G., Dong Z., Luo W., Feng Y., Wu B., Yang W. (2014). Gravel morphometric analysis based on digital images of different gobi surfaces in northwestern China. J. Desert Res..

[9] Ding J., Li Z., Zhang H., Zhang P., Cao X., Feng Y. (2022). Quantifying the aboveground biomass (AGB) of Gobi Desert Shrub communities in Northwestern China based on unmanned aerial vehicle (UAV) RGB images. Land.

[10] Ma X., Wang X. (2021). Aboveground and belowground biomass and its’ allometry for Salsola passerina shrub in degraded steppe desert in Northwestern China. Land Degrad. Dev..

[11] Tian Y., Zhang Q., Huang H., Huang Y., Tao J., Zhou G., Zhang Y., Yang Y., Lin J. (2022). Aboveground biomass of typical invasive mangroves and its distribution patterns using UAV-LiDAR data in a subtropical estuary: Maoling River estuary, Guangxi, China. Ecol. Indic..

[12] Abella S.R. (2020). Cover–biomass relationships of an invasive annual grass, Bromus rubens, in the Mojave Desert. Invasive Plant Sci. Manag..

[13] Yao Z., Qing H., Yang L., Zhao L. (2021). Non-destructive aboveground biomass estimation of Leymus chinensis individual across large scale. Ecol. Indic..

[14] Jin L., Li X., Huang Q., Yang H., Huang J. (2022). Allometry, biomass and productivity in *Pinus massoniana* forests of China: An updated review of published data. Pol. J. Ecol..

[15] Penderis C.A., Kirkman K.P. (2014). Using partial volumes to estimate available browse biomass in Southern African semi-arid savannas. Appl. Veg. Sci..

[16] Xu K., He L., Hu H., Wang Z., Lin M., Liu S., Du Y., Li Y., Wang G. (2019). Indirect effects of water availability in driving and predicting productivity in the Gobi desert. Sci. Total Environ..

[17] Greaves H.E., Vierling L.A., Eitel J.U., Boelman N.T., Magney T.S., Prager C.M., Griffin K.L. (2016). High-resolution mapping of aboveground shrub biomass in Arctic tundra using airborne lidar and imagery. Remote Sens. Environ..

[18] Wang Z., Bastin G.N., Liu L., Caccetta P.A., Peng D. (2015). Estimating woody above-ground biomass in an arid zone of central Australia using Landsat imagery. J. Appl. Remote Sens..

[19] Zandler H., Brenning A., Samimi C. (2015). Quantifying dwarf shrub biomass in an arid environment: Comparing empirical methods in a high dimensional setting. Remote Sens. Environ..

[20] Cho M.-S., Roy D.P., Kashongwe H.B., Yan L., Shen M. (2025). Assessment and improvement of GEDI canopy height estimation in tropical and temperate forests. Sci. Remote Sens..

[21] Smith W.K., Dannenberg M.P., Yan D., Herrmann S., Barnes M.L., Barron-Gafford G.A., Biederman J.A., Ferrenberg S., Fox A.M., Hudson A. (2019). Remote sensing of dryland ecosystem structure and function: Progress, challenges, and opportunities. Remote Sens. Environ..

[22] Tucker C., Brandt M., Hiernaux P., Kariryaa A., Rasmussen K., Small J., Igel C., Reiner F., Melocik K., Meyer J. (2023). Sub-continental-scale carbon stocks of individual trees in African drylands. Nature.

[23] Chen W., Zhao J., Cao C., Tian H. (2018). Shrub biomass estimation in semi-arid sandland ecosystem based on remote sensing technology. Glob. Ecol. Conserv..

[24] Xie D., Huang H., Feng L., Sharma R.P., Chen Q., Liu Q., Fu L. (2023). Aboveground biomass prediction of arid shrub-dominated community based on airborne LiDAR through parametric and nonparametric methods. Remote Sens..

[25] Riihimäki H., Luoto M., Heiskanen J. (2019). Estimating fractional cover of tundra vegetation at multiple scales using unmanned aerial systems and optical satellite data. Remote Sens. Environ..

[26] Alonzo M., Andersen H.-E., Morton D.C., Cook B.D. (2018). Quantifying boreal forest structure and composition using UAV structure from motion. Forests.

[27] Näsi R., Viljanen N., Kaivosoja J., Alhonoja K., Hakala T., Markelin L., Honkavaara E. (2018). Estimating biomass and nitrogen amount of barley and grass using UAV and aircraft based spectral and photogrammetric 3D features. Remote Sens..

[28] Cunliffe A.M., Anderson K., Boschetti F., Brazier R.E., Graham H.A., Myers-Smith I.H., Astor T., Boer M.M., Calvo L.G., Clark P.E. (2022). Global application of an unoccupied aerial vehicle photogrammetry protocol for predicting aboveground biomass in non-forest ecosystems. Remote Sens. Ecol. Conserv..

[29] Zhang X., Zhang F., Qi Y., Deng L., Wang X., Yang S. (2019). New research methods for vegetation information extraction based on visible light remote sensing images from an unmanned aerial vehicle (UAV). Int. J. Appl. Earth Obs. Geoinf..

[30] Freestone F.L., Brown P., Campbell C.J., Wood D.B., Nielsen D.L., Henderson M.W. (2017). Return of the lignum dead: Resilience of an arid floodplain shrub to drought. J. Arid. Environ..

[31] McCann J.A., Keith D.A., Kingsford R.T. (2022). Measuring plant biomass remotely using drones in arid landscapes. Ecol. Evol..

[32] Avtar R., Suab S.A., Syukur M.S., Korom A., Umarhadi D.A., Yunus A.P. (2020). Assessing the influence of UAV altitude on extracted biophysical parameters of young oil palm. Remote Sens..

[33] Hu P., Guo W., Chapman S.C., Guo Y., Zheng B. (2019). Pixel size of aerial imagery constrains the applications of unmanned aerial vehicle in crop breeding. ISPRS J. Photogramm. Remote Sens..

[34] Santos Santana L., Araújo E Silva Ferraz G., Bedin Marin D., Dienevam Souza Barbosa B., Mendes Dos Santos L., Ferreira Ponciano Ferraz P., Conti L., Camiciottoli S., Rossi G. (2021). Influence of flight altitude and control points in the georeferencing of images obtained by unmanned aerial vehicle. Eur. J. Remote Sens..

[35] Mao P., Jiang B., Shi Z., He Y., Shen T., Qiu G.Y. (2023). Effects of UAV flight height on biomass estimation of desert shrub communities. Ecol. Indic..

[36] Mao P., Ding J., Jiang B., Qin L., Qiu G.Y. (2022). How can UAV bridge the gap between ground and satellite observations for quantifying the biomass of desert shrub community?. ISPRS J. Photogramm. Remote Sens..

[37] Zhao Y., Liu X., Wang Y., Zheng Z., Zheng S., Zhao D., Bai Y. (2021). UAV-based individual shrub aboveground biomass estimation calibrated against terrestrial LiDAR in a shrub-encroached grassland. Int. J. Appl. Earth Obs. Geoinf..

[38] Bojórquez A., Martínez-Yrízar A., Búrquez A., Jaramillo V.J., Mora F., Balvanera P., Álvarez-Yépiz J.C. (2020). Improving the accuracy of aboveground biomass estimations in secondary tropical dry forests. For. Ecol. Manag..

[39] Kouamé Y.A.G., Millan M., N’Dri A.B., Charles-Dominique T., Konan M., Bakayoko A., Gignoux J. (2022). Multispecies allometric equations for shrubs and trees biomass prediction in a Guinean savanna (West Africa). Silva Fenn..

[40] Nyamjav J., Batsaikhan M.E., Li G., Li J., Luvsanjamba A., Jin K., Xiao W., Wu L., Indree T., Qin A. (2020). Allometric equations for estimating above-ground biomass of *Nitraria sibirica* Pall. in Gobi Desert of Mongolia. PLoS ONE.

[41] Siebert S., Gries D., Zhang X., Runge M., Buerkert A. (2004). Non-destructive dry matter estimation of Alhagi sparsifolia vegetation in a desert oasis of Northwest China. J. Veg. Sci..

[42] Tao Y., Zhang Y. (2013). Multi-scale biomass estimation of desert shrubs: A case study of Haloxylon ammodendron in the Gurbantunggut Desert, China. Acta Prataculturae Sin..

[43] Kenzo T., Himmapan W., Yoneda R., Tedsorn N., Vacharangkura T., Hitsuma G., Noda I. (2020). General estimation models for above-and below-ground biomass of teak (*Tectona grandis*) plantations in Thailand. For. Ecol. Manag..

[44] Nyamukuru A., Whitney C., Tabuti J.R.S., Esaete J., Low M. (2023). Allometric models for aboveground biomass estimation of small trees and shrubs in African savanna ecosystems. Trees For. People.

[45] Conti G., Gorne L.D., Zeballos S.R., Lipoma M.L., Gatica G., Kowaljow E., Whitworth-Hulse J.I., Cuchietti A., Poca M., Pestoni S. (2019). Developing allometric models to predict the individual aboveground biomass of shrubs worldwide. Glob. Ecol. Biogeogr..

[46] Huang C., Feng C., Ma Y., Liu H., Wang Z., Yang S., Wang W., Fu S., Chen H.Y. (2022). Allometric models for aboveground biomass of six common subtropical shrubs and small trees. J. For. Res..

[47] Ma J., Yuan C., Zhou J., Li Y., Gao G., Fu B. (2021). Logistic model outperforms allometric regression to estimate biomass of xerophytic shrubs. Ecol. Indic..

[48] Yue J., Guan J., Deng L., Zhang J., Li G., Du S. (2018). Allocation pattern and accumulation potential of carbon stock in natural spruce forests in northwest China. PeerJ.

[49] Conti G., Enrico L., Casanoves F., Díaz S. (2013). Shrub biomass estimation in the semiarid Chaco forest: A contribution to the quantification of an underrated carbon stock. Ann. For. Sci..

[50] Sileshi G.W. (2014). A critical review of forest biomass estimation models, common mistakes and corrective measures. For. Ecol. Manag..

[51] Yao X., Yang G., Wu B., Jiang L., Wang F. (2021). Biomass estimation models for six shrub species in hunshandake sandy land in inner Mongolia, Northern China. Forests.

[52] Yang H., Wang Z., Tan H., Gao Y. (2017). Allometric models for estimating shrub biomass in desert grassland in northern China. Arid Land Res. Manag..

[53] Matsuo T., Martínez-Ramos M., Onoda Y., Bongers F., Lohbeck M., Poorter L. (2024). Light competition drives species replacement during secondary tropical forest succession. Oecologia.

[54] McGregor S., Ismail A.-L., Duker R., Liversage W., Maharaj S., Mills A.J., Van Mazijk R., Butynski C., Schutgens M., Schleicher M. (2025). Allometric equations for hyper-arid desert plant species of AlUla County, Saudi Arabia. Carbon Balance Manag..

[55] Zeng H., Liu Q., Feng Z., Ma Z. (2010). Biomass equations for four shrub species in subtropical China. J. For. Res..

[56] Ali A., Xu M.-S., Zhao Y.-T., Zhang Q.-Q., Zhou L.-L., Yang X.-D., Yan E.-R. (2015). Allometric biomass equations for shrub and small tree species in subtropical China. Silva Fenn..

[57] Djomo A.N., Ibrahima A., Saborowski J., Gravenhorst G. (2010). Allometric equations for biomass estimations in Cameroon and pan moist tropical equations including biomass data from Africa. For. Ecol. Manag..

[58] Evangelista P., Kumar S., Stohlgren T.J., Crall A.W., Newman G.J. (2007). Modeling aboveground biomass of Tamarix ramosissima in the Arkansas River basin of southeastern Colorado, USA. West. N. Am. Nat..

[59] Enquist B.J., West G.B., Charnov E.L., Brown J.H. (1999). Allometric scaling of production and life-history variation in vascular plants. Nature.

[60] Niklas K.J. (2004). Modelling Below- and Above-ground Biomass for Non-woody and Woody Plants. Ann. Bot..

[61] Daba D.E., Soromessa T. (2019). The accuracy of species-specific allometric equations for estimating aboveground biomass in tropical moist montane forests: Case study of Albizia grandibracteata and Trichilia dregeana. Carbon Balance Manag..

[62] Levin S.A. (1992). The problem of pattern and scale in ecology: The Robert H. MacArthur award lecture. Ecology.

[63] Moorcroft P.R., Hurtt G.C., Pacala S.W. (2001). A method for scaling vegetation dynamics: The ecosystem demography model (ED). Ecol. Monogr..

[64] Fang J., Chen A., Peng C., Zhao S., Ci L. (2001). Changes in forest biomass carbon storage in China between 1949 and 1998. Science.

[65] Jagodziński A.M., Dyderski M.K., Gęsikiewicz K., Horodecki P., Cysewska A., Wierczyńska S., Maciejczyk K. (2018). How do tree stand parameters affect young Scots pine biomass?–Allometric equations and biomass conversion and expansion factors. For. Ecol. Manag..

[66] Cai T., Ju C., Yang X. (2009). Comparison of ridge regression and partial least squares regression for estimating above-ground biomass with landsat images and terrain data in mu us sandy land, China. Arid Land Res. Manag..

[67] Fei C., Dong Y.Q., An S.Z. (2022). Factors driving the biomass and species richness of desert plants in northern Xinjiang China. PLoS ONE.

[68] Tao Y., Zhang Y., Downing A. (2013). Similarity and difference in vegetation structure of three desert shrub communities under the same temperate climate but with different microhabitats. Bot. Stud..

[69] Tyagi S., Tyagi P.K., Kumar A., Yadav R.K. (2026). Artificial Intelligence and Digital Technologies for Smart Farming and Precision Agriculture. Phytores. Tech..

[70] Li Y., Wu H., Blaschke T., Pradhan B., Mohamed Shariff A.R.B., Avtar R., Lin A., Shao W. (2026). Integrating future land use uncertainty and spatial scales into ecosystem service degradation risk assessment for urban ecological management. Appl. Geogr..

[71] Zhang Z., Dong Z., Li J., Qian G., Jiang C. (2016). Implications of surface properties for dust emission from gravel deserts (gobis) in the Hexi Corridor. Geoderma.

[72] Gao J., Shi Y., Zhang H., Chen X., Zhang W., Shen W., Xiao T., Zhang Y. (2022). China Regional 250m Fractional Vegetation Cover Data Set (2000–2023).

[73] Zhong B., Wang X. (2025). A 30-m Aboveground Biomass Dataset for China in 2020.

